# Identification of a Mutation Associated with Fatal Foal Immunodeficiency Syndrome in the Fell and Dales Pony

**DOI:** 10.1371/journal.pgen.1002133

**Published:** 2011-07-07

**Authors:** Laura Y. Fox-Clipsham, Stuart D. Carter, Ian Goodhead, Neil Hall, Derek C. Knottenbelt, Paul D. F. May, William E. Ollier, June E. Swinburne

**Affiliations:** 1 Animal Health Trust, Newmarket, United Kingdom; 2 Department of Infection Biology, School of Veterinary Science, University of Liverpool, Liverpool, United Kingdom; 3 Centre for Genomic Research, Institute of Integrative Biology, University of Liverpool, Liverpool, United Kingdom; 4 Department of Veterinary Clinical Science, Equine Hospital, University of Liverpool, Liverpool, United Kingdom; 5 Townhead Veterinary Centre, Townhead Farm, Penrith, United Kingdom; 6 Centre for Integrated Genomic Medical Research, University of Manchester, Manchester, United Kingdom; Cornell University, United States of America

## Abstract

The Fell and Dales are rare native UK pony breeds at risk due to falling numbers, in-breeding, and inherited disease. Specifically, the lethal Mendelian recessive disease Foal Immunodeficiency Syndrome (FIS), which manifests as B-lymphocyte immunodeficiency and progressive anemia, is a substantial threat. A significant percentage (∼10%) of the Fell ponies born each year dies from FIS, compromising the long-term survival of this breed. Moreover, the likely spread of FIS into other breeds is of major concern. Indeed, FIS was identified in the Dales pony, a related breed, during the course of this work. Using a stepwise approach comprising linkage and homozygosity mapping followed by haplotype analysis, we mapped the mutation using 14 FIS–affected, 17 obligate carriers, and 10 adults of unknown carrier status to a ∼1 Mb region (29.8 – 30.8 Mb) on chromosome (ECA) 26. A subsequent genome-wide association study identified two SNPs on ECA26 that showed genome-wide significance after Bonferroni correction for multiple testing: BIEC2-692674 at 29.804 Mb and BIEC2-693138 at 32.19 Mb. The associated region spanned 2.6 Mb from ∼29.6 Mb to 32.2 Mb on ECA26. Re-sequencing of this region identified a mutation in the sodium/myo-inositol cotransporter gene (*SLC5A3)*; this causes a P446L substitution in the protein. This gene plays a crucial role in the regulatory response to osmotic stress that is essential in many tissues including lymphoid tissues and during early embryonic development. We propose that the amino acid substitution we identify here alters the function of SLC5A3, leading to erythropoiesis failure and compromise of the immune system. FIS is of significant biological interest as it is unique and is caused by a gene not previously associated with a mammalian disease. Having identified the associated gene, we are now able to eradicate FIS from equine populations by informed selective breeding.

## Author Summary

Foal Immunodeficiency Syndrome (FIS) is a genetic disease that affects two related British pony breeds, namely the Fell and the Dales. Foals with FIS appear to be normal at birth but within a few weeks develop evidence of infection such as diarrhoea, pneumonia, etc. The infections are resistant to treatment, and the foals die or are euthanized before three months of age. The foals also suffer from a severe progressive anemia. Being a recessive condition, the disease is difficult to control without a diagnostic DNA test to identify symptom-free carrier parents. Within the last few years the horse genome has been sequenced, and this has allowed the development of tools to identify genetic mutations in the horse at high resolution. In this article we demonstrate the use of these new tools to identify the location of the FIS mutation. The presumptive causal lesion was then identified by sequencing this region. This has enabled us to develop a test that can be used to identify carrier ponies, allowing breeders to avoid FIS in their foal crop.

## Introduction

The Fell and Dales are related sturdy pony breeds traditionally used as pack animals to carry goods over the difficult upland terrain of northern England. Both breeds experienced near extinction during WWII, and the current populations are descended from very few animals. It is likely that this genetic bottleneck, together with the use of prominent sires, was responsible for the emergence of a fatal Mendelian recessive disease, FIS, which currently affects up to 10% of Fell and 1% of Dales foals (data from UK breed societies). Both of these breeds are registered with the Rare Breeds Survival Trust due to their falling numbers, and important position in the UK's agricultural heritage.

FIS was first described in 1998 as a unique syndrome in which affected Fell foals develop diarrhoea, cough and fail to suckle [1]. Despite an initial response to treatment, the infections persist and were shown to be due to a primary B-cell deficiency [2] associated with reduced antibody production, with tested immunoglobulin isotypes including IgM, IgGa, IgGb and IgG(T) being significantly reduced [3]. Paradoxically, circulating T-lymphocyte numbers are normal [4]. The reduced antibody levels in affected foals are consistent with an inability to generate an adaptive immune response, resulting in immunodeficiency once colostrum-derived immunoglobulin levels decrease at 3–6 weeks of age. This loss of maternally derived antibodies correlates with typical onset of FIS signs at 4–6 weeks. Concurrently, affected foals develop a non-hemolytic, non-regenerative progressive profound anemia [5], in itself severe enough to cause death and the main marker for euthanasia decisions by vets.

As a result of FIS, foals die or are humanely destroyed between 1–3 months of age, the disease being 100% fatal. In 2009, this condition was reported in the Dales breed [6]; it is likely that the mutation has passed between the breeds given the similarity between them and the practice of occasional interbreeding. The clinical and pathological findings for FIS are compatible with a primary defect of genetic origin [1], and this is supported by extensive genealogical studies [6], [7]. FIS has a pattern of inheritance typical of an autosomal recessive disease, and the likely founder animal, which features in both the Fell and Dales studbooks, has been traced by pedigree analysis.

Primary immunodeficiences, which include depleted levels of lymphocytes and/or immunoglobulins, have previously been reported in the horse. The recessive defect ‘severe combined immunodeficiency’ (SCID), which is found in the Arabian breed, comprises a fatal deficiency in T- and B-lymphocyte numbers and function. The underlying lesion was found to be a 5 base-pair deletion in the gene coding DNA-dependent kinase, catalytic subunit DNA-PK_CS_ [8], a protein involved in V(D)J recombination required for adaptive immunity [9]. Like FIS foals, SCID foals have a markedly reduced thymus and have reduced numbers of germinal centers in secondary lymphoid organs [10]; unlike SCID foals however, FIS foals have apparently normal numbers of circulating T-cells [4]. Primary aggamaglobinemia is rare in horses and comprises of a complete absence of immunoglobulin and reduced peripheral B-lymphocyte levels, with normal T-lymphocyte activity [11]. In this respect there is a similarity to FIS, however primary aggamaglobinemia is only observed in males and is X-linked. Furthermore, profound anemia in combination with B-lymphopenia has not previously been reported in the horse or any other species, and as such FIS appears to be a unique disease process.

Here we report the mapping and identification of the genetic lesion that causes FIS. An initial scan using microsatellite markers identified the chromosome region responsible. The opportune production of a SNP chip, which utilized the SNP variants generated during the sequencing of the equine genome (http://www.broadinstitute.org/mammals/horse) then allowed a confirmatory association scan. This was followed by re-sequencing of the implicated region in order to identify the causal mutation.

## Results

### Microsatellite Analysis

A genome-wide microsatellite scan was performed on 41 individuals taken from five pedigrees of Fell ponies in which FIS was segregating (Figure S1), using a panel of 228 markers (Table S1). The data were examined both for loss of heterozygosity and for linkage (Table S2). Only one microsatellite, at 30.25 Mb on ECA26, showed a significant loss of heterozygosity (*χ* ^2^ = 7.15, *P* = 0.028) and significant linkage (LOD score  = 3.29 at θ = 0) to the disease.

### SNP Association Scan

**Figure 1:**
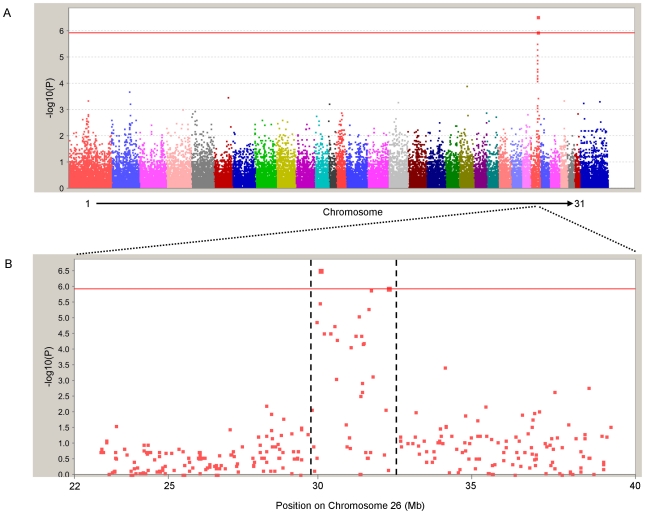
Identification of locus responsible for FIS by genome-wide association scan. (A) Manhattan plot of association test results showing the chromosome locations of the 42,536 SNPs which passed quality control against –log_10_ *(P).* The red line indicates the threshold for genome-wide significance after Bonferroni correction for multiple testing (*P*_raw_ = 1.2×10^−6^), which corresponds to an alpha value of 0.05. (B) Focus on region of ECA26 that shows FIS association. The vertical broken lines indicate the region (29.6–32.2 Mb) which was investigated further by fine-mapping.

The location of the lesion was confirmed and refined using a genome-wide association analysis with an equine SNP array (Illumina EquineSNP50 Infinium BeadChip), which contains 54,602 validated SNPs. After applying quality control (see Materials and Methods), data were available for 42,536 SNPs in 49 individuals (18 FIS-affected and 31 controls). To consider whether there was any population stratification among the samples, a multi-dimensional scaling plot of the genome-wide identity-by-state distances was performed (Figure S2); there was no significant difference between the affected and control samples for the first two components (*P* = 0.553). In addition, a quantile-quantile plot (Figure S3) to compare the expected and observed distributions of –log_10_(*P*), obtained by a basic association test, showed that there was little evidence of inflation of the test statistics (genomic inflation factor λ = 1.04), indeed the test statistics appear to be marginally depressed rather than inflated. No correction was considered necessary. Two SNPs on ECA26 showed genome-wide significance after Bonferroni correction for multiple testing (Figure 1A). These are BIEC2-692674 at 29.804 Mb (*P*_raw_ = 2.88×10^−7^) and BIEC2-693138 at 32.19 Mb (*P*_raw_ = 1.08×10^−6^). The associated region spanned 2.6 Mb from position 29.6 Mb to 32.2 Mb on ECA26 (Figure 1B).

**Figure 2:**
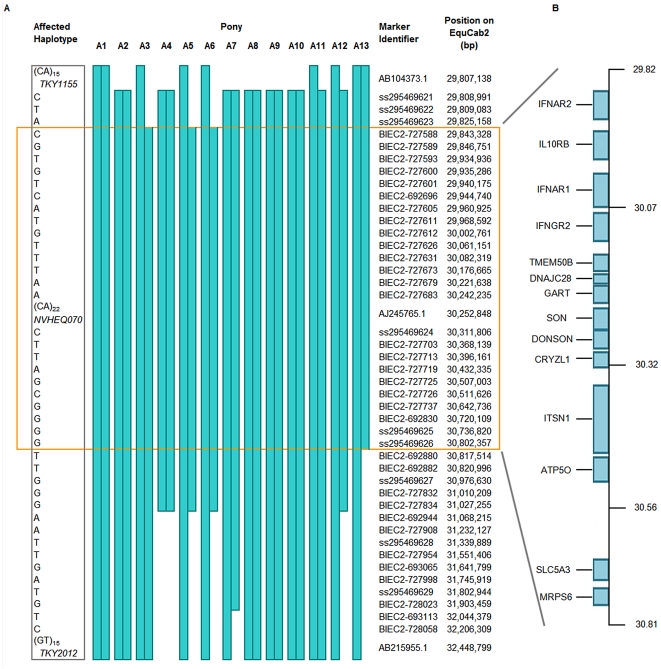
SNP haplotypes and genes from the region of ECA26 genetically linked and associated with FIS. (A) The affected SNP haplotype is shown on the left. The affected alleles for three key microsatellite markers are also included. The extent of conserved affected haplotypes present in the 13 affected individuals (A1–13) are indicated with blue bars. Accession numbers (newly identified SNPs) or the local SNP ID number (http://www.broadinstitute.org/ftp/distribution/horse_snp_release/v2/), together with the genome position are given. The minimal 992 kb shared region of homozygosity (29,825 – 30,817 kb) is out-lined in orange. (B) The positions of the 14 ENSEMBL annotated genes within the conserved block are indicated.

In a subsequent fine-mapping phase, 62 additional SNPs within the region were genotyped on 13 FIS-affected samples. Several novel SNPs were identified (dbSNP ss295469621-295469629). In addition, two further microsatellites were also genotyped (Table S1). The homozygous affected haplotype was shared by these animals over a 992 kb segment (Figure 2A). According to ENSEMBL gene prediction, fourteen genes lie in this interval (Figure 2B).

### Resequencing of Critical Region

Five selected individual animals were re-sequenced over this critical region using sequence capture by NimbleGen arrays followed by GS FLX Titanium sequencing (GenBank submission under study accession no. ERP000492). The five individuals comprised one affected foal (A13 on Figure 2), the two obligate carrier parents, one apparently clear animal and one obligate carrier chosen for maximal homozygosity across the region. The FIS carrier status and familial relationships were confirmed for each individual by parentage verification. The last animal proved particularly useful in eliminating many potential causal variants. In total, eight verified SNPs were identified in the affected foal, narrowing the critical region to 375,063 bp (ECA26: 30,372,557 – 30,747,620 bp). Coverage of this critical interval was increased from 92.9% to 98.4% using Sanger sequencing; none of the remaining gaps fell within 200 bp of protein-coding sequence. Only one variant, a SNP at 30,660,224 bp, segregated as expected for a causal recessive mutation within the five sequenced samples. In addition there was no evidence of DNA rearrangement, duplication or insertion/deletion seen in the affected foal (Figure S4). The segregating SNP was assessed for validity as an FIS marker in equine populations.

### Population Screen

Subsequently all 38 available affected foals (37 Fell, 1 Dales) were shown to be homozygous for the affected allele and all 21 available obligate carriers were heterozygous. A selection of Fell and Dales samples which were submitted to the Animal Health Trust for parentage verification between 2000 and 2010 were anonymously screened for the affected allele: 82 / 214 (38%) of the Fells and 16 / 87 (18%) of the Dales were heterozygous for the lesion and no homozygous affecteds were discovered. These carrier rates are consistent with the approximate observed disease prevalence of 10% in the Fell and 1% in the Dales populations. In addition, a selection of horse breeds (184 individuals from 11 breeds consisting of Thoroughbred (n = 29), Appaloosa (n = 8), Arab (n = 21), Warmblood Sport Horse (n = 17), Lipizzaner (n = 2), Cleveland Bay (n = 20), Dartmoor Pony (n = 19), Icelandic Horse (n = 8), New Forest Pony (n = 20), Sheltand Pony (n = 20) and Shire (n = 20)) which were considered unlikely to have interbred with either the Fell or Dales was genotyped and all proved homozygous wild-type.

## Discussion

The identification of a mutation that segregates 100% with the disease has enabled a diagnostic test to be developed and offered to breeders and owners, allowing them to avoid carrier-carrier matings, and consequently drastically reduce the numbers of FIS-affected foals born each year. A gradual reduction in the use of carrier animals will, over time, lead to a reduction in the affected allele frequency in the population, while conserving the gene pool as much as possible. In addition, other equine breeds that may have interbred with the Fell or Dales will now be screened for FIS carriers.

**Figure 3:**
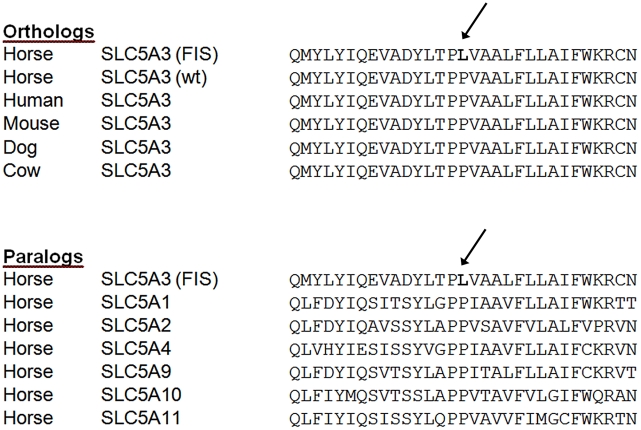
SLC5A3 amino acid sequences alignments in the region of the FIS mutation. The amino acid affected by the mutation, indicated with an arrow, is conserved in all placental mammals now sequenced with high coverage (n = 11); the top panel shows alignments of this gene region in a selection of these mammals. The bottom panel shows alignments of this region with other members of the SLC5 gene family in the horse which show structural similarity; the amino acid affected by the mutation is conserved in all of these members.

The FIS-associated SNP falls within the single exon of the sodium/myo-inositol co-transporter gene (*SLC5A3*, also known as *SMIT*), which is a cell membrane transporter protein responsible for the co-transport of sodium ions and myo-inositol. This SNP is non-synonymous, causing a P446L substitution in SLC5A3; this amino acid residue (equivalent residue 451 in the human protein) is conserved in all 11 placental mammals for which high-coverage sequence is now available (selection shown in Figure 3). Similarly, this residue is conserved in other solute carrier family 5 (SLC5) paralogs in the horse which share similar structural homology (Figure 3).

The crystal structure of a bacterial homolog of SLC5A1 (sodium/glucose co-transporter 1) has recently been elucidated [12] and shows this member of the SLC5 family to have 14 transmembrane helices; the structural conformations adopted during transport, and the precise positions of substrate binding during transfer are now being identified [13]. Alignment of the protein sequences of the SLC5 family suggests that P446 in equine SLC5A3 is located in a transmembrane helix which is involved in forming the substrate cavity [12] and which tilts during substrate transfer. The two prolines at positions 445 and 446 may be required for effective substrate binding by closing the substrate binding site after the substrate is bound. Proline residues introduce structural destabilisation into alpha helices and have long been obvious candidates for points of conformational change required for substrate binding and release [14]. Indeed, replacement of prolines in the transmembrane helices of transport proteins has shown that some residues are profoundly important in transport, affecting either substrate affinity or substrate movement [15].

*SLC5A3* is an osmotic stress response gene, which acts to prevent dehydration caused by increased osmotic pressure in the extracellular environment. Dehydration causes the disruption of numerous cellular functions by denaturation of intracellular molecules and damage to sub-cellular architecture [16]. Extreme osmotic conditions are found in the kidney, although osmotic response mechanisms have also been found in numerous tissues, and in particular are critical for lymphocyte development and function [17], [18].

The mechanism by which the osmotic stress response is mediated in mammals is not completely understood, but involves a signaling cascade comprising Rho-type small G-proteins, p38 Mitogen-activated protein kinase (p38MAPK) and the transcription factor, Nuclear Factor of Activated T-cells 5 (NFAT5) [19]. NFAT5 directly stimulates the transcription of hyperosmolarity-responsive genes, of which *SLC5A3* is one. These act to counterbalance the effects of extracellular osmotic pressure by transporting small organic osmolytes, such as myo-inositol, into the cell, thereby maintaining isotonicity with respect to extra-cellular conditions [20]. NFAT5 is the only known transcriptional activator of hyperosmolarity response genes, and was shown to be essential for normal lymphocyte proliferation and adaptive immunity [18]. Targeted knockout of *NFAT5* in mice results in late gestational lethality whereas partial loss of function leads to defects in adaptive immunity and a substantially reduced spleen and thymus [18]. Furthermore, transgenic studies identify loss of T-cell mediated immunity as the prime deficiency ensuing from aberrant NFAT5 activity [21], [22]. Similarly FIS-affected foals have markedly reduced thymus and spleen with a lack of germinal centers [1], [23]. However, FIS disease immunopathology indicates that FIS foals have apparently normal circulating T-cell numbers with only peripheral blood B-lymphocyte numbers significantly depleted; currently, there are no data available indicating NFAT5 activity in specific B-lymphocyte functions.

Studies are now required to demonstrate the functional differences between the Pro446 and Leu446 forms of the protein. This will be achieved by introducing this mutation into transgenic mice and assessing transport function. Further investigation into the physiological consequences of this mutation will then also be possible. In particular, it will be important to identify how the mutation leads to profound anemia and B-lymphopenia whilst neutrophils and T-cell numbers (including CD4/CD8 ratio) and function (responses to mitogens PHA and Con A) appear normal [4]. Importantly, it must be investigated whether there is a defect in T-cell function that is currently undetected or whether the antigen presenting function of B-cells is so suppressed that the T-cells cannot respond. In addition, it must be noted that the lymphoid organs in FIS foals have depleted thymus tissue and poor germinal centre development in spleen and lymph nodes, which suggests that there may be some unidentified T-cell dysfunction. Alternatively, any T-cell defects could be due to severe inflammatory responses in these very sick foals.

SLC5A3 is not associated with any described mammalian disease, although a role in the pathogenicity of Down Syndrome is suggested [24]. The effect of loss of SLC5A3 activity has not been comprehensively studied, however *SLC5A3* knockout mice die shortly after birth due to hypoventilation [25], probably due to failure of the peripheral nervous system [26]; similarly FIS-affected foals have peripheral ganglionopathy [1].

There is relatively little literature on SLC5A3 function in hemopoietic or immunological tissues, in any species, although a role for osmotic control in developing cells is likely. Due to uncertainty regarding tissue distribution and function of SLC5A3, it cannot be assumed that the profound anemia and severe loss of circulating B-lymphocytes in FIS is directly due to a functional change in SLC5A3 expression or function; formal proof that this is the case will entail functional studies. Whilst there is no doubt that the mutation in this gene is predictive of carrier or disease status, the mechanism by which this amino acid change could lead to the two described pathologies is speculative. It is, of course possible that the mutation site is close to another, as yet unidentified, mutation that is ultimately responsible for the severe hematological and immunological changes in homozygotes. However, all coding sequence within the critical region has been fully investigated and this is the only variant that segregates with the disease. We hypothesize that the phenotype seen in FIS-affected foals is either a result of partial loss or subtle alteration in SLC5A3 activity that has deleterious effects on B-lymphocyte and erythroid development but cannot discount the involvement of other genetic variants in the critical region. Whichever is the case, further analysis of FIS is justified as this genetically determined combination of immune phenotypes has not previously been reported in any other species.

## Materials and Methods

### Ethics Statement

Procedures were limited to the collection of blood by jugular venipuncture or hairs pulled from the mane or tail. Blood samples were taken as veterinary diagnostic procedures as all study animals were equine patients presenting with clinical signs suggestive of FIS or were healthy related or unrelated animals that were blood tested for anemia and/or B-lymphocyte deficiency.

### Study Population and Diagnostic Procedures

Many of the Fell and Dales ponies used in this study have been described previously [6], [7]. Study animals were all equine patients presenting with clinical signs suggestive of FIS or were healthy related or unrelated Fell or Dales ponies that were blood tested for anemia and/or B-lymphocyte deficiency. Several FIS foals presented subsequent to euthanasia. Pedigree information was available for many of the Fell ponies (Figure S1), and these samples (n = 41) were used in the linkage and homozygosity mapping analysis. An additional ten samples were added to these for the association study; these were isolated samples for which no pedigree information and/or samples from immediate family were available. Any adult Fell pony was eligible as a control for the association study.

FIS diagnosis was based on breed, age of animal (4–8 weeks at presentation), and profound anemia with no other predisposing cause, and was confirmed on pathology. Specifically, this indicated severely reduced numbers (or absence) of germinal centers in spleen and regional lymph nodes. B-lymphocyte deficiency was also used for FIS diagnosis. Many accompanying clinical signs were also reported, primarily related to opportunistic infections, but these were not considered diagnostic alone.

### Samples

Blood samples were collected in EDTA collection tubes from all of the Fell pony individuals indicated in Figure S1, and from a Dales foal and it's parents [6]. Genomic DNA was isolated from the samples using a Nucleon™ BACC Genomic DNA Extraction Kit.

### Microsatellite Markers

A panel of 228 markers, distributed as evenly as possible over the equine genome and described in Table S1, was used. Two further markers, TKY1155 and TKY2012, which were located in the implicated region, were subsequently genotyped.

The genome scan was performed in multiplexes of three markers. Four PCR reactions, each utilising a different fluorescent dye, were pooled together post-PCR to form a panel of 12 markers for analysis. An 18 bp tail (5′-TGACCGGCAGCAAAATTG-3′) was added to the 5′ end of the forward primer and a complementary fluorescent labelling primer was included in the PCR reaction as a means of making the reactions more efficient and to reduce costs [27]. Amplification was performed in 6 µl volumes, using 2.5 pmol of reverse, 1 pmol of tailed-forward, 5 pmol of the labelled universal primer (either 6-FAM, VIC, NED, or PET), 20 ng genomic DNA, 0.75 unit AmpliTaq Gold (Applied Biosystems), 1× GeneAmp PCR buffer II (Applied Biosystems), 1.5 mM MgCl2, and 200 µM each dNTP. After denaturation at 94°C for 10 min, a 30-cycle PCR of 94°C for 1 min, 55°C for 1 min, and 72°C for 1 min, followed by 8 cycles of 94°C for 1 min, 50°C for 1 min, and 72°C for 1 min was performed, followed by a final extension at 72°C for 30 min. Genotyping analysis was performed on an ABI3100 (Applied Biosystems) according to the manufacturer's instructions. Genotyping data was analysed with GeneMapper version 4.0 (Applied Biosystems); alleles were assigned to pre-defined bins and automatically given an appropriate integer value. Mendelian inheritance was checked.

### Linkage and Homozygosity Mapping

We used 41 ponies (14 FIS-affected, 17 obligate carriers, 10 adults of unknown carrier status) for which pedigree information and DNA was available, in the linkage analysis (Figure S1).

A parametric linkage analysis was carried out using SUPERLINK v.1.5 [28] assuming an autosomal recessive mode of inheritance. The disease allele frequency was estimated at 0.1, with 100% penetrance.

Pearson's chi^2^ test of independence was used to identify markers where homozygosity varied significantly between the cases and controls. An A×2 (where A =  number of alleles at a given locus) contingency table with A-1 degrees of freedom was used. Expected and observed heterozygosity values were computed for cases and controls for all markers exhibiting a positive LOD score, using ARLEQUIN [29]. Statistical significance was assessed by calculating the one-tailed probability of the chi squared distribution.

### Genome-Wide Association Mapping

SNP genotyping on 51 genomic DNA samples was performed using standard manufacturer's protocols by Cambridge Genomic Services (University of Cambridge, UK). The Illumina EquineSNP50 Infinium BeadChip, which contains 54,602 validated SNPs, was used; information on this array is available at http://www.illumina.com/documents/products/datasheets/datasheet_equine_snp50.pdf. Quality control and genotype calling were performed using GenomeStudio v.2009.2 (Illumina Inc.). Samples with a call rate <95% were discarded (n = 2). We performed a basic case-control association analysis on the remaining 49 samples (18 affected and 31 controls). Analysis was performed with the software package PLINK [30]. SNPs with low minor allele frequency (<0.02) or genotyping rate (<90%) were excluded; this left 42,536 SNPs for analysis. The presence of population stratification was assessed using multi-dimensional scaling (Figure S2) and quantile-quantile plots were drawn to confirm that there was no over-inflation of the test statistics (Figure S3).

### Fine Mapping

A total of 62 polymorphic SNPs were studied in 13 affected individuals. A subset of these helped to delineate recombination breakpoints and these are identified in Figure 2. Information regarding these SNPs can be found at http://www.broadinstitute.org/ftp/distribution/horse_snp_release/v2/equcab2.0_chr26_snps.xls.

PCR amplification of the target sequence containing each informative SNP was performed in 12 µl volumes containing 20 ng genomic DNA, 0.75 unit AmpliTaq Gold, 1× GeneAmp PCR buffer II, 1.5 mM MgCl_2_, 200 µM each dNTP, 10 pmol of reverse and of tailed-forward primer. A PCR program of 94°C for 10 min, followed by 30 cycles of 94°C for 1 min, 58°C for 1 min, and 72°C for 2 min, and then an extension of 72°C for 10 min was used. The PCR products were purified (MultiScreen PCR_96_ filter plates; Millipore) before sequencing in a 6 µl volume using 0.5 µl of 5× BigDye Terminator v3.1 (Applied Biosystems), 5–20 ng PCR template, 1 µl of 1× BigDye sequencing buffer and 3.2 pmol universal sequencing primer (Sigma-Aldrich). Templates >500 bp were also sequenced in the reverse direction. Sequencing was performed using cycle sequencing: 96°C for 0.5 min, 44 cycles of 92°C for 4 s, 58°C for 4 s and 72°C for 1.5 min. Purification was performed by isopropanol precipitation followed by sequencing on an ABI3100 according to the manufacturer's instructions. Sequences were viewed using STADEN [31].

### Re-Sequencing of Candidate Region

This was performed at the Centre for Genomic Research (University of Liverpool, UK). A region of 3 Mb (ECA26: 28,942,655 – 31,942,655 Mb) was selected for re-sequencing which encompassed the critical region. Custom tiling 385 k NimbleGen Sequence Capture arrays (http://www.454.com/products-solutions/experimental-design-options/nimblegen-sequence-capture.asp) which covered 92.9% of the target were designed from the horse reference sequence using standard repeat-masking algorithms. Five individuals were selected for re-sequencing consisting of one affected pony (A13 in Figure 2), its parents, one obligate carrier selected for maximal homozygosity over the region and one individual apparently homozygous wild-type.

Sequencing was performed using GS FLX Titanium Series chemistry and assembled using Roche Newbler software v2.0.00. An average 34-fold read depth was obtained. Sequence from each of the five sequenced animals was aligned to the EquCab2 reference sequence using the Artemis Comparison Tool (ACT) [32] to identify possible rearrangements or insertion/deletions (Figure S4).

### Identifying Candidate Mutations for FIS

MySQL (Oracle Corporation) was used to interrogate the data. The critical region was narrowed using heterozygous variants in the affected foal and Sanger sequencing subsequently verified these. The narrowed critical region was then interrogated for variants that segregated as expected for a recessive mutation; putative causal variants were confirmed or disproved using Sanger sequencing.

## Supporting Information

Figure S1 The Fell pony pedigrees used for linkage analysis. Affected (FIS) individuals are shown shaded in black and obligate carriers are indicated with a dot. Individuals that are neither affected or obligate carriers are shown un-shaded. Individuals also coloured yellow were genotyped and used for linkage and homozygosity mapping. Double lines indicate consanguinity.
(TIF)

Figure S2 Multidimensional scaling plot of first two components. Multidimensional scaling analysis illustrating the first two components of Identity-By-State similarity for all FIS-affected (red squares) and controls (blue circles) used in the genome-wide association analysis. A permutation test (10,000 permutations) for between-group IBS differences showed that there was no significant difference between the affected and controls (*P* = 0.553).
(TIF)

Figure S3 Observed versus expected log_10_(*P*). A Q-Q plot showing the distribution of expected versus observed –log_10_ *P* for the basic association test with no adjustment. The pink diagonal shows the values expected under the null hypothesis. The observed –log_10_ *P* values match the expected values along the major portion of the graph and deviate towards the end illustrating the small number of true associations. The plot indicates minimal population stratification and therefore no corrections were subsequently made to the data.
(TIF)

Figure S4 Alignment of re-sequenced region from FIS pony to the EquCab2 reference sequence. Sequence from the FIS-affected pony was aligned to the EquCab2 reference sequence using the Artemis Comparison Tool (ACT) [32] to identify possible rearrangements, duplications or insertion/deletions. Red indicates alignment of the sequence. White regions indicate missing sequence caused either by missing probe design, or small tandem repeats which result in reads stacking on top of each other and causing a break in the contig. Blue lines indicate inverted repeats scattered throughout the sequence. Black lines represent blocks of synteny used for sequence comparison and do not represent sequence variation. These alignments provide no evidence for significant rearrangement, duplication or insertion/deletion within the sequences; there is an excellent match between the FIS pony and the reference sequence.
(TIF)

Table S1 Panel of 228 microsatellite markers used in linkage and homozygosity mapping.
(DOC)

Table S2 Linkage analysis and homozygosity mapping of FIS using a genome-wide microsatellite set.
(DOC)
