# Supplementary figures and images for: Identification of a Mutation Associated with Fatal Foal Immunodeficiency Syndrome in the Fell and Dales Pony
Source: PLoS Genet. 2011 Jul 7;7(7):e1002133. doi: 10.1371/journal.pgen.1002133 (PMC3131283; doi:10.1371/journal.pgen.1002133)

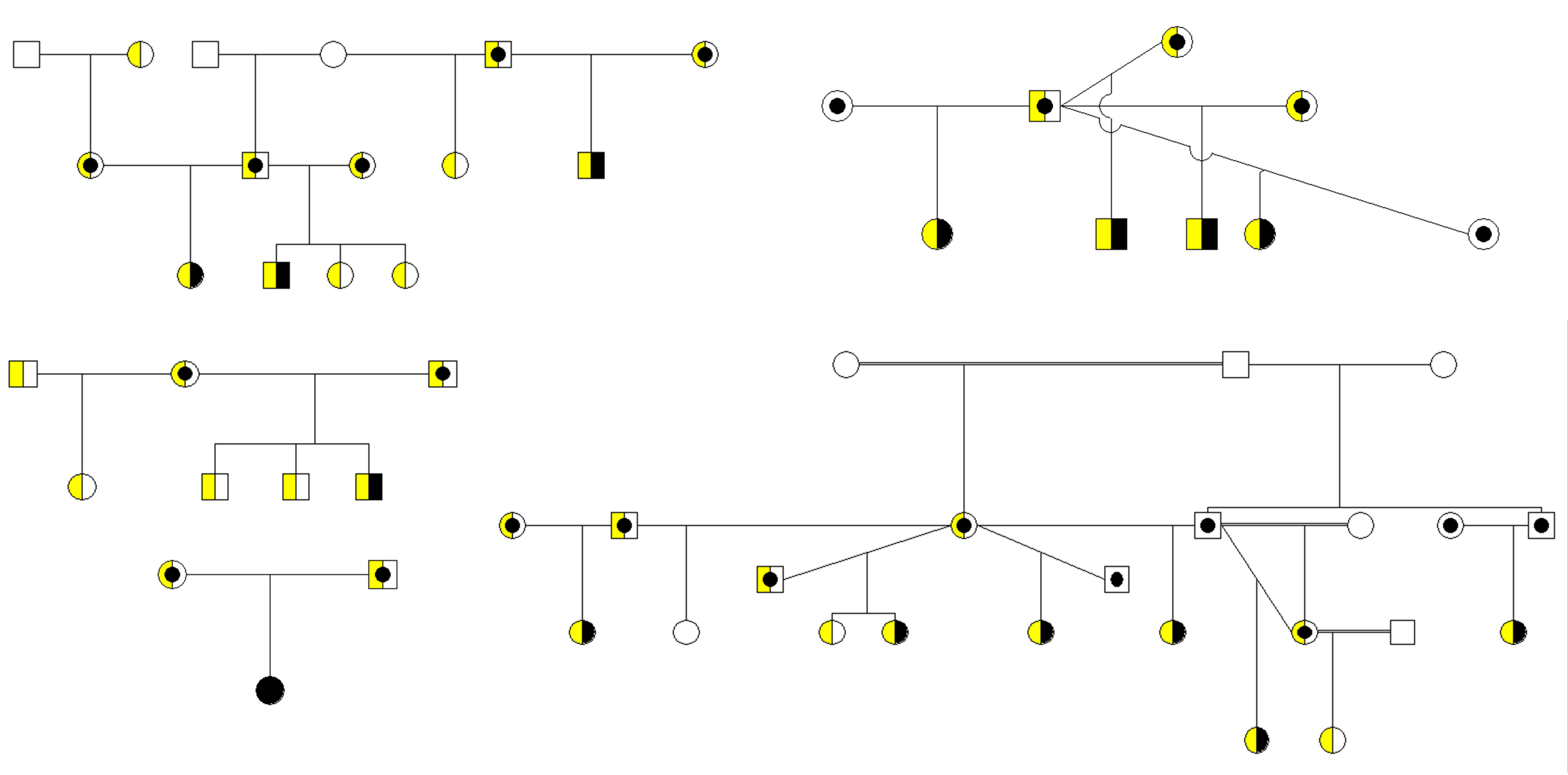

Supplement: Figure S1 — The Fell pony pedigrees used for linkage analysis. Affected (FIS) individuals are shown shaded in black and obligate carriers are indicated with a dot. Individuals that are neither affected or obligate carriers are shown un-shaded. Individuals also coloured yellow were genotyped and used for linkage and homozygosity mapping. Double lines indicate consanguinity. (TIF) [file pgen.1002133.s001.tif]

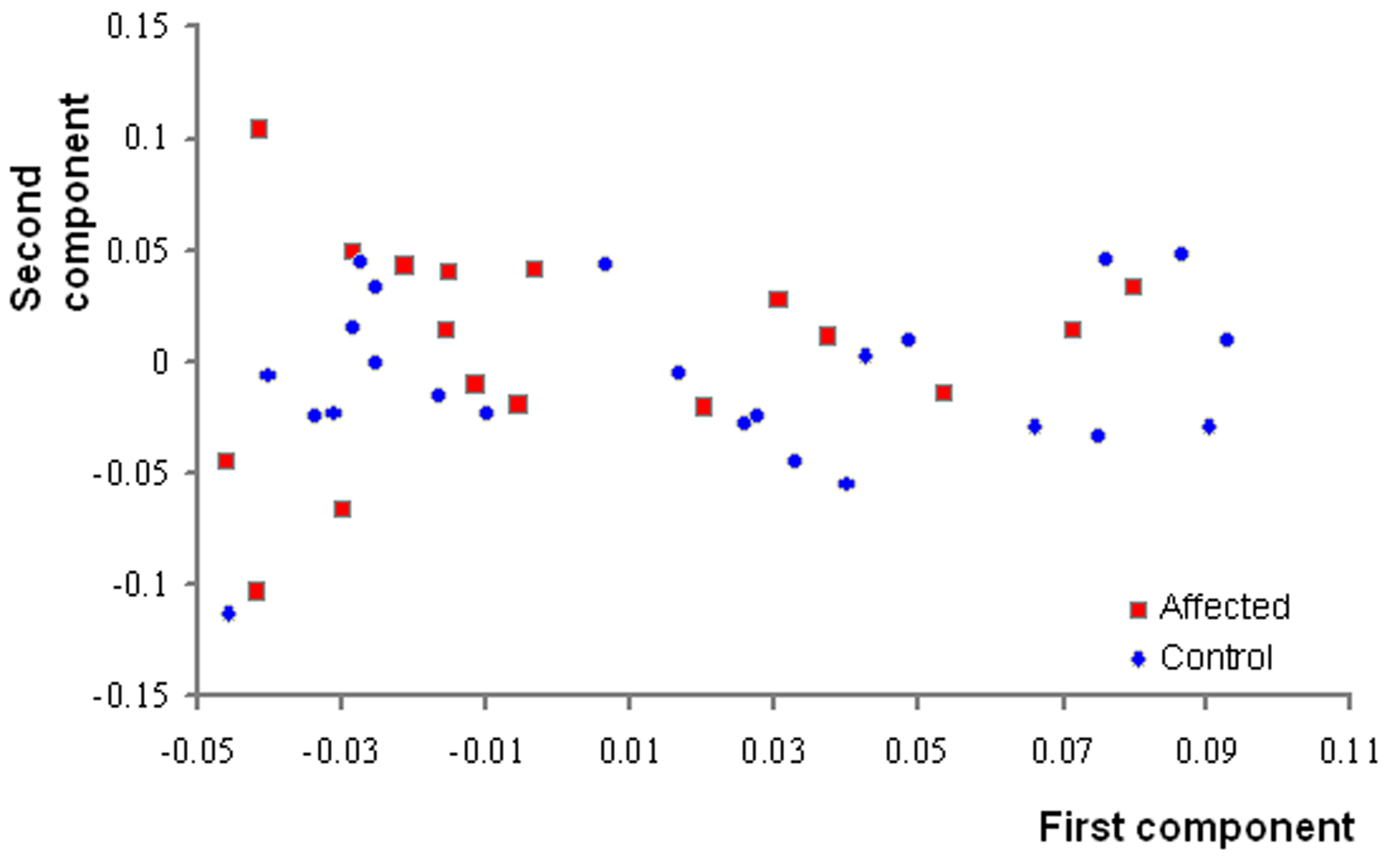

Supplement: Figure S2 — Multidimensional scaling plot of first two components. Multidimensional scaling analysis illustrating the first two components of Identity-By-State similarity for all FIS-affected (red squares) and controls (blue circles) used in the genome-wide association analysis. A permutation test (10,000 permutations) for between-group IBS differences showed that there was no significant difference between the affected and controls (P = 0.553). (TIF) [file pgen.1002133.s002.tif]

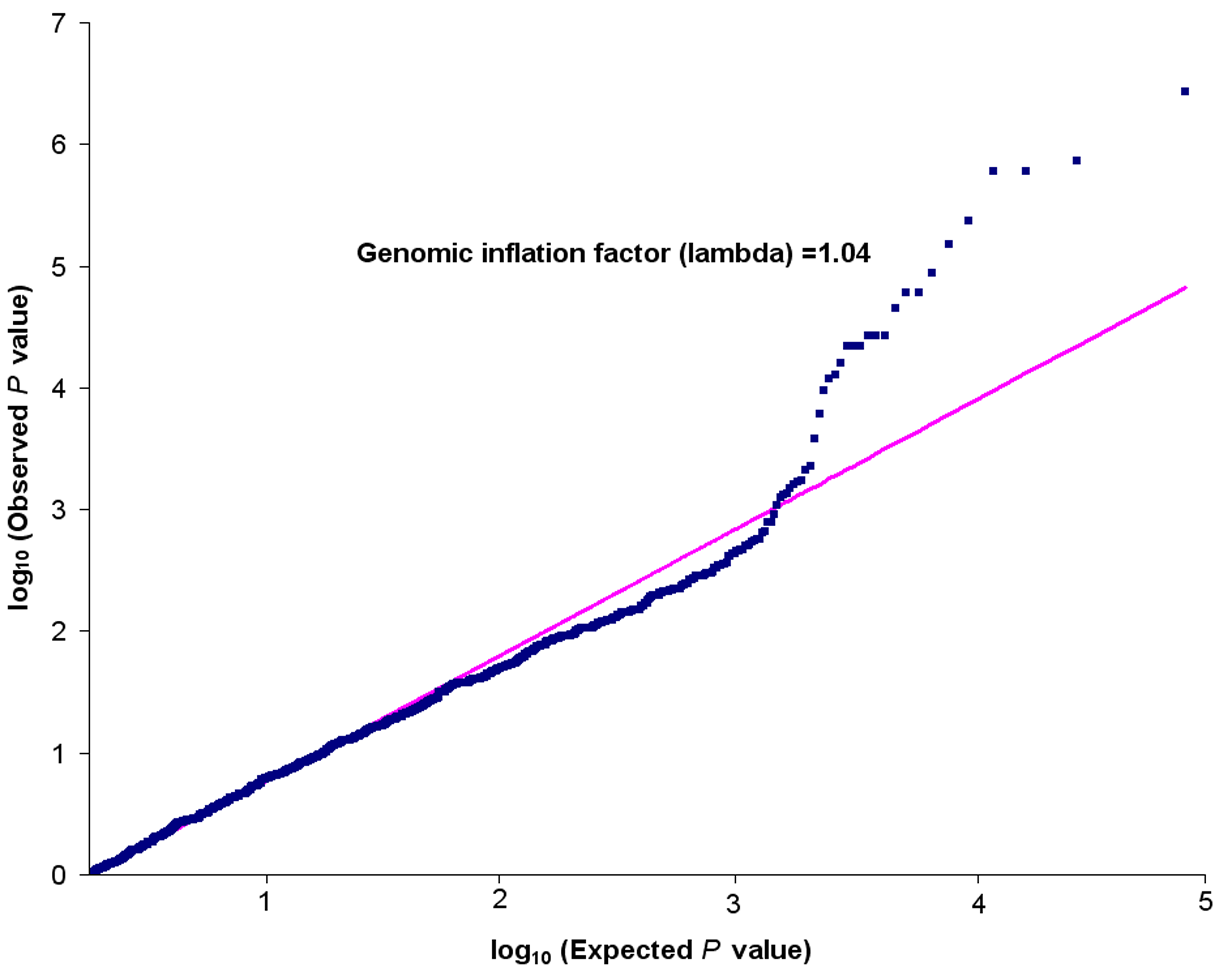

Supplement: Figure S3 — Observed versus expected log10(P). A Q-Q plot showing the distribution of expected versus observed –log10 P for the basic association test with no adjustment. The pink diagonal shows the values expected under the null hypothesis. The observed –log10 P values match the expected values along the major portion of the graph and deviate towards the end illustrating the small number of true associations. The plot indicates minimal population stratification and therefore no corrections were subsequently made to the data. (TIF) [file pgen.1002133.s003.tif]

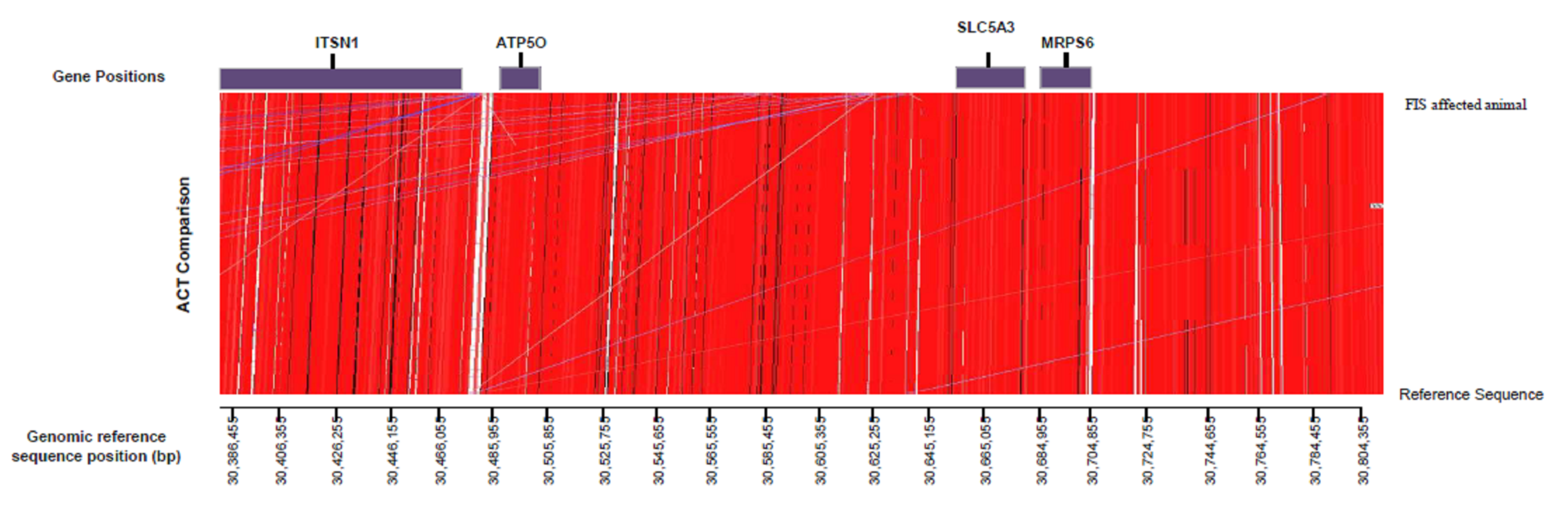

Supplement: Figure S4 — Alignment of re-sequenced region from FIS pony to the EquCab2 reference sequence. Sequence from the FIS-affected pony was aligned to the EquCab2 reference sequence using the Artemis Comparison Tool (ACT) [32] to identify possible rearrangements, duplications or insertion/deletions. Red indicates alignment of the sequence. White regions indicate missing sequence caused either by missing probe design, or small tandem repeats which result in reads stacking on top of each other and causing a break in the contig. Blue lines indicate inverted repeats scattered throughout the sequence. Black lines represent blocks of synteny used for sequence comparison and do not represent sequence variation. These alignments provide no evidence for significant rearrangement, duplication or insertion/deletion within the sequences; there is an excellent match between the FIS pony and the reference sequence. (TIF) [file pgen.1002133.s004.tif]
