# Supplementary material for: Identification of a Mutation Associated with Fatal Foal Immunodeficiency Syndrome in the Fell and Dales Pony
Source: PLoS Genet. 2011 Jul 7;7(7):e1002133. doi: 10.1371/journal.pgen.1002133 (PMC3131283; doi:10.1371/journal.pgen.1002133)
Supplement: Table S1 — Panel of 228 microsatellite markers used in linkage and homozygosity mapping. (DOC) [file pgen.1002133.s005.doc]

| **Marker Name** | **ECA** | **Position on genome reference sequence (EquCab2) as identified by BLAT (Mb)** | **Primer sequences (unpublished markers)** |
| --- | --- | --- | --- |
| HLM005 | 1 | 1.63 |  |
| HP27 | 1 | 14.34 | F : TGTTCATTCAACCATTCTGCCC |
|  |  |  | R : AAACCCTCCACTACCCCATTCC |
| ASB041 | 1 | 18.27 |  |
| HMS059 | 1 | 26.31 |  |
| AHT026 | 1 | 41.04 |  |
| COR100 | 1 | 50.78 |  |
| UCDEQ487 | 1 | 66.49 |  |
| TKY015 | 1 | 71.24 |  |
| SGCV002 | 1 | 76.35 |  |
| AHT040 | 1 | 89.89 |  |
| UM041 | 1 | 91.61 |  |
| LEX077 | 1 | 95.69 |  |
| ASB008 | 1 | 99.98 |  |
| 1CA043 | 1 | 110.28 |  |
| 1CA025 | 1 | 117.76 |  |
| AHT058 | 1 | 127.64 |  |
| TKY295 | 1 | 153.15 |  |
| 1CA016 | 1 | 157.18 |  |
| COR006 | 1 | 161.49 |  |
| HMS007 | 1 | 162.38 |  |
| COR053 | 1 | 182.81 |  |
| ASB018 | 2 | 5.26 |  |
| TKY384 | 2 | 9.00 |  |
| TKY003 | 2 | 22.74 |  |
| AHT035 | 2 | 23.35 |  |
| HMS054 | 2 | 28.46 |  |
| AHT067 | 2 | 30.94 |  |
| HMS051 | 2 | 32.98 |  |
| TKY340 | 2 | 39.27 |  |
| UMNe129 | 2 | 64.17 |  |
| A14 | 2 | 74.47 |  |
| NVHEQ224 | 2 | 76.04 | F: GTGACATGGCCTTCTATCC |
|  |  |  | R: CTAACTTGGCATTCCCTTTC |
| UM076 | 2 | 87.06 |  |
| TKY798 | 2 | 93.96 |  |
| AHT064 | 2 | 100.81 |  |
| VHL123 | 2 | 109.82 |  |
| COR026 | 2 | 117.18 |  |
| COR028 | 3 | 11.07 |  |
| COR033 | 3 | 13.47 |  |
| AHT022 | 3 | 21.13 |  |
| AHT090 | 3 | 31.62 |  |
| LEX057 | 3 | 36.31 |  |
| ASB023 | 3 | 79.28 |  |
| LEX007 | 3 | 86.98 |  |
| AHT097 | 3 | 99.04 |  |
| UMNe192 | 4 | 3.13 |  |
| HMS006 | 4 | 7.23 |  |
| TKY223 | 4 | 8.65 |  |
| LEX050 | 4 | 49.36 |  |
| TKY552 | 4 | 65.15 |  |
| TKY375 | 4 | 72.56 |  |
| TKY363 | 4 | 96.44 |  |
| AHT042 | 4 | 107.02 |  |
| NVHEQ102 | 5 | 21.78 | F : CAACTGGGCCTCAATCTTGG |
|  |  |  | R : AGGGTTGGGGTCATCATCC |
| TKY271 | 5 | 25.66 |  |
| HMS052 | 5 | 28.65 |  |
| AHT066 | 5 | 28.73 |  |
| AHT050 | 5 | 44.29 |  |
| UMNe255 | 5 | 53.68 |  |
| LEX034 | 5 | 76.17 |  |
| ASB010 | 5 | 78.29 |  |
| LEX014 | 5 | 88.96 |  |
| TKY344 | 5 | 92.50 |  |
| UMNe237 | 5 | 97.46 |  |
| UMNe177 | 6 | 11.26 |  |
| TKY312 | 6 | 17.32 |  |
| UMNe233 | 6 | 19.98 |  |
| HMS055 | 6 | 33.74 |  |
| TKY377 | 6 | 36.03 |  |
| TKY360 | 6 | 42.22 |  |
| COR070 | 6 | 65.85 |  |
| TKY323 | 6 | 69.22 |  |
| NVHEQ232 | 7 | 14.70 | F : CACAGGAAAGATGTAAAAGG |
|  |  |  | R : GGAGAAACTGATGAACAACC |
| LEX038 | 7 | 28.96 |  |
| TKY005 | 7 | 43.60 |  |
| TKY338 | 7 | 64.77 |  |
| TKY282 | 7 | 69.27 |  |
| TKY613 | 7 | 89.95 |  |
| COR097 | 8 | 1.44 |  |
| UMNe504 | 8 | 7.10 |  |
| UM034 | 8 | 18.88 |  |
| LEX023 | 8 | 25.94 |  |
| SGCV032 | 8 | 57.50 |  |
| TKY359 | 8 | 64.17 |  |
| COR003 | 8 | 64.25 |  |
| HTG004 | 9 | 1.50 |  |
| AHT104 | 9 | 12.15 |  |
| COR008 | 9 | 18.91 |  |
| TKY291 | 9 | 30.03 |  |
| AHT053 | 9 | 51.32 |  |
| ASB004 | 9 | 61.73 |  |
| UMNe212 | 9 | 73.29 |  |
| AHT049 | 10 | 0.41 |  |
| HMS023 | 10 | 2.71 |  |
| TKY131 | 10 | 10.00 | F : GGTAGGTAGTGTCAGAATCGAGC |
|  |  |  | R : CATCTCTACCGCAAGTGAAACCAG |
| AHT015 | 10 | 12.76 |  |
| LEX017 | 10 | 28.68 |  |
| HMS002 | 10 | 52.71 |  |
| UMNe265 | 10 | 55.79 |  |
| NVHEQ067 | 10 | 71.40 |  |
| AHT086 | 10 | 76.70 |  |
| AHT044 | 11 | 4.13 |  |
| NVHEQ040 | 11 | 9.11 |  |
| UMNe240 | 11 | 11.74 |  |
| SGCV024 | 11 | 19.54 |  |
| TKY010 | 11 | 39.68 |  |
| TKY276 | 11 | 47.15 |  |
| UMNe191 | 12 | 10.83 |  |
| COR058 | 12 | 27.95 |  |
| UCDEQ497 | 12 | 32.57 |  |
| UMNe145 | 13 | 14.08 |  |
| VHL047 | 13 | 16.89 |  |
| SERCA1 | 13 | 19.91 |  |
| TKY371 | 13 | 25.65 |  |
| UMNe187 | 14 | 9.66 |  |
| TKY267 | 14 | 12.08 |  |
| LEX043 | 14 | 16.14 |  |
| UM010 | 14 | 25.47 |  |
| UMNe234 | 14 | 40.32 |  |
| TKY491 | 14 | 81.18 |  |
| UMNe239 | 14 | 91.64 |  |
| TKY383 | 15 | 2.39 |  |
| UMNe222 | 15 | 7.05 |  |
| UMNe198 | 15 | 17.65 |  |
| TKY369 | 15 | 29.10 |  |
| UMNe156 | 15 | 41.33 |  |
| ASB002 | 15 | 54.61 |  |
| COR077 | 15 | 61.82 |  |
| HMS001 | 15 | 85.45 |  |
| COR075 | 15 | 86.78 |  |
| AHT038 | 16 | 30.27 |  |
| TKY349 | 16 | 33.48 |  |
| AHT014 | 16 | 57.79 |  |
| ASB042 | 16 | 68.01 |  |
| COR064 | 16 | 70.27 |  |
| HMS058 | 16 | 81.91 |  |
| TKY373 | 17 | 4.23 |  |
| AHT102 | 17 | 7.60 |  |
| COR032 | 17 | 41.43 |  |
| LEX067 | 17 | 59.86 |  |
| TKY379 | 17 | 64.74 |  |
| AHT098 | 17 | 76.81 |  |
| TKY019 | 18 | 0.54 |  |
| LEX016 | 18 | 14.88 |  |
| UMNe050 | 18 | 23.06 |  |
| TKY692 | 18 | 36.96 |  |
| TKY322 | 18 | 54.22 |  |
| UMNe501 | 18 | 63.50 |  |
| TKY016 | 18 | 66.84 |  |
| UMNe243 | 18 | 78.93 |  |
| AHT041 | 19 | 0.09 |  |
| ASB007 | 19 | 10.77 |  |
| HTG024 | 19 | 18.22 |  |
| LEX073 | 19 | 24.40 |  |
| TKY372 | 19 | 31.01 |  |
| ASB011 | 19 | 45.15 |  |
| AHT055 | 19 | 53.49 |  |
| AHT018 | 20 | 10.05 |  |
| LEX064 | 20 | 15.30 |  |
| TKY136 | 20 | 27.07 | F : CAGGGTTCATCCAAAGACTC |
|  |  |  | R : GCTGAAGGAGGTGAAATAGG |
| UMNe056 | 20 | 29.29 |  |
| UM011 | 20 | 33.51 |  |
| TKY273 | 20 | 44.76 |  |
| UMNe151 | 20 | 49.18 |  |
| HMS042 | 20 | 63.74 |  |
| SGCV014 | 21 | 1.61 |  |
| TKY678 | 21 | 3.30 |  |
| UMNe229 | 21 | 11.55 |  |
| COR073 | 21 | 20.25 |  |
| LEX060 | 21 | 20.30 |  |
| TKY280 | 21 | 31.97 |  |
| AHT077 | 21 | 42.95 |  |
| AHT010 | 21 | 48.32 | F : CTCCTAGTACCATTTTTGAAAC |
|  |  |  | R : CAAAGTAGAGCAAGACTGGC |
| TKY306 | 21 | 53.42 |  |
| TKY806 | 21 | 53.66 |  |
| HTG014 | 22 | 14.28 |  |
| TKY785 | 22 | 23.09 |  |
| HTG021 | 22 | 27.35 |  |
| COR107 | 22 | 37.60 | F : TCTCAAGTATTGATTTTGGGG |
|  |  |  | R : TGCAGAAGCTATTTCTGGTG |
| HMS047 | 22 | 39.95 |  |
| AHT031 | 22 | 45.99 |  |
| SGCV019 | 22 | 47.78 |  |
| COR060 | 23 | 3.25 |  |
| AHT072 | 23 | 13.12 |  |
| TKY346 | 23 | 15.44 |  |
| HP13 | 23 | 28.34 | F : CCTAAGCACTGCCTTCAGCATC |
|  |  |  | R : GATCATCACTCCATATCCACTCCC |
| UM022 | 23 | 32.97 |  |
| TKY385 | 23 | 37.22 |  |
| COR084 | 23 | 40.40 |  |
| TKY269 | 23 | 45.10 |  |
| UMNe159 | 23 | 47.98 |  |
| UM012 | 24 | 16.14 |  |
| TKY357 | 24 | 26.36 |  |
| UMNe210 | 24 | 29.97 |  |
| TKY394 | 24 | 33.98 |  |
| COR024 | 24 | 41.00 |  |
| UCDEQ467 | 24 | 42.76 |  |
| AHT075 | 24 | 43.00 |  |
| UCDEQ464 | 25 | 1.98 |  |
| COR018 | 25 | 15.69 |  |
| TKY316 | 25 | 25.73 |  |
| UMNe066 | 26 | 14.18 |  |
| LEX044 | 26 | 19.65 |  |
| UM005 | 26 | 26.04 |  |
| *TKY1155 | 26 | 29.8 | F: TATCAGCTCAGGGCGAATCT |
|  |  |  | R: AAACCTGGGCATCTTCCTTT |
| *TKY2012 | 26 | 32.44 | F: AGGTGTTATGGGCCATCC |
|  |  |  | R: CCTCAGATGGGTGACAAAAT |
| NVHEQ070 | 26 | 30.25 |  |
| TKY437 | 27 | 1.58 |  |
| COR040 | 27 | 17.15 |  |
| TKY315 | 27 | 20.77 |  |
| TKY288 | 27 | 26.15 |  |
| AHT082 | 27 | 27.27 |  |
| VHL150 | 27 | 29.65 |  |
| COR017 | 27 | 35.28 |  |
| UM003 | 28 | 10.56 |  |
| TKY320 | 28 | 25.54 |  |
| UMNe166 | 28 | 30.19 |  |
| TKY299 | 28 | 33.87 |  |
| TKY364 | 28 | 40.09 |  |
| UCDEQ425 | 28 | 43.09 |  |
| COR082 | 29 | 4.28 |  |
| TKY628 | 29 | 18.05 |  |
| ASB043 | 29 | 30.34 |  |
| LEX025 | 30 | 2.04 |  |
| HTG027 | 30 | 7.29 |  |
| UMNe530 | 30 | 11.73 |  |
| UCDEQ455 | 30 | 27.41 |  |
| TKY368 | 31 | 4.21 |  |
| TKY274 | 31 | 11.46 |  |
| TKY278 | 31 | 21.42 |  |

The details for all published markers can be found in GenBank, while primer sequences for all unpublished markers are listed.

*Markers added during fine mapping stage
