# Supplementary material for: Identification of a Mutation Associated with Fatal Foal Immunodeficiency Syndrome in the Fell and Dales Pony
Source: PLoS Genet. 2011 Jul 7;7(7):e1002133. doi: 10.1371/journal.pgen.1002133 (PMC3131283; doi:10.1371/journal.pgen.1002133)
Supplement: Table S2 — Linkage analysis and homozygosity mapping of FIS using a genome-wide microsatellite set. (DOC) [file pgen.1002133.s006.doc]

| **Marker** | **ECA** | **Mb** | **LOD score at various recombination fractions between marker and disease locus** | | | | | **Observed Heterozygosity** | | **Chi square test of independence** | | |
| --- | --- | --- | --- | --- | --- | --- | --- | --- | --- | --- | --- | --- |
|  |  |  | **0** | **0.01** | **0.1** | **0.1** | **0.2** | **Control** | **FIS Affected** | **Chi Square** | **d.f.** | ***P* value** |
| HLM005 | 1 | 1.63 | 1.91 | 1.87 | 1.7 | 1.5 | 1 | 0.462 | 0.125 | 1.177 | 1 | 0.278 |
| SGCV002 | 1 | 76.35 | 1 | 0.96 | 0.8 | 0.6 | 0.4 | 0.154 | 0.125 | 0 | 1 | 1 |
| VHL123A | 2 | 109.82 | 2.97 | 2.9 | 2.6 | 2.2 | 1.5 | 0.154 | 0.063 | 2.518 | 2 | 0.284 |
| TKY223 | 4 | 8.65 | -5.3 | -0.3 | 0.8 | 1.1 | 1 | 0.654 | 0.558 | 3.785 | 4 | 0.436 |
| LEX014 | 5 | 88.96 | -2.4 | -0.2 | 0.8 | 1 | 1 | 0.741 | 0.714 | 1.334 | 3 | 0.721 |
| UM237 | 5 | 97.46 | 0.39 | 0.81 | 1.2 | 1.1 | 0.6 | 0.702 | 0.744 | 1.782 | 3 | 0.619 |
| TKY312 | 6 | 17.32 | 0.67 | 0.78 | 1 | 1 | 0.8 | 0.556 | 0.667 | 2.029 | 3 | 0.566 |
| TKY005 | 7 | 43.6 | 1.27 | 1.25 | 1.2 | 1 | 0.7 | 0.192 | 0.188 | 0.277 | 2 | 0.87 |
| TKY131 | 10 | 10 | 0.26 | 0.47 | 0.9 | 1.1 | 1 | 0.692 | 0.438 | 6.265 | 6 | 0.394 |
| UCDEQ497 | 12 | 32.57 | 1.38 | 1.36 | 1.3 | 1.1 | 0.7 | 0.154 | 0.125 | 1.774 | 2 | 0.412 |
| UM010 | 14 | 25.47 | -0.2 | 0.87 | 1.4 | 1.5 | 1.1 | 0.692 | 0.375 | 3.708 | 6 | 0.716 |
| TKY491 | 14 | 81.18 | -1.2 | 0.28 | 1.2 | 1.3 | 1 | 0.577 | 0.563 | 1.023 | 2 | 0.6 |
| HMS001 | 15 | 85.45 | 1.33 | 1.31 | 1.2 | 1.1 | 0.7 | 0.154 | 0.438 | 3.782 | 5 | 0.581 |
| AHT014 | 16 | 57.79 | 1.22 | 1.18 | 1.1 | 0.9 | 0.6 | 0.192 | 0 | 2.644 | 2 | 0.267 |
| HMS058 | 16 | 81.91 | 0.17 | 0.71 | 1.3 | 1.5 | 1.2 | 0.385 | 0.375 | 2.31 | 4 | 0.679 |
| UM022 | 23 | 32.97 | 2.59 | 2.55 | 2.3 | 2 | 1.2 | 0.5 | 0.25 | 5.454 | 4 | 0.244 |
| TKY394 | 24 | 33.98 | -0.6 | 1.56 | 2.1 | 2.1 | 1.5 | 0.462 | 0.313 | 3.227 | 3 | 0.358 |
| *TKY1155 | 26 | 29.81 | 1.68 | 1.7 | 1.6 | 1.5 | 1 | 0.577 | 0.375 | 0.166 | 3 | 0.983 |
| NVHEQ070 | 26 | 30.25 | **3.29** | **3.2** | 2.8 | 2.4 | 1.6 | 0.462 | 0.063 | 7.15 | 2 | **0.028** |
| AHT082 | 27 | 27.27 | 1.81 | 1.91 | 1.9 | 1.7 | 1.2 | 0.269 | 0.313 | 5.36 | 3 | 0.147 |
| UMNE530 | 30 | 11.73 | 1.35 | 1.42 | 1.4 | 1.3 | 0.9 | 0.462 | 0.125 | 3.032 | 3 | 0.387 |

The initial genome scan was performed on 44 ponies taken from five pedigrees (see Figure S1) using a panel of 228 markers. Only markers which demonstrated a LOD score > 1 are shown (20/228). Parameters for the analyses are described in the Methods. Only one marker (NVHEQ070 on ECA26) demonstrated a significant LOD score or a significant P value in the Chi square test, and these results are shaded.

*Subsequently one further marker from ECA26 (TKY1155) was also genotyped
